# Annotations

**Published:** 1894-06-02

**Authors:** 


### Annotations.

### The British Medical Benevolent Fund.

It is a serious pity that the British Medical Benevolent Fund is obliged to report a falling off in its annual subscriptions of not less than a sixth of their full amount. In 1892 the subscriptions reached a total of £1,261, whilst in 1893 only £1,024 were raised. This will be melancholy reading for a very large number of widows and aged and enfeebled daughters of deceased medical men. The report just published tells us—and we know from our own knowledge how true it is—that there is nothing but this small fund between a great many doctors' widows and starvation or the workhouse. The fund gives to some of these old ladies annuities; to others grants. But we are let into the secret of the depths of their poverty when we are told that the average amount of the annuity is £20. Educated women in scores, and perhaps hundreds, grateful, profoundly grateful, for £20 a-year! It is a saddening thought. But apparently even this will have to be made smaller. For we are told that whilst the subscription list is diminishing, the number of applicants is increasing. Can nothing more be done? Surely it can! A few doctors who have fortunes should remember the fund more liberally, and more doctors should certainly let their names appear on the subscription list. There are over thirty-one thousand medical men in the United Kingdom, and not more than thirteen hundred of them subscribe to this admirable fund. Twenty-nine thousand of our doctors must be either very poor or very mean. Perhaps they are only careless. If so, we hope the officials of the fund will give them a reminder such as they cannot put aside.

### Some Justice for Doctors.

The action of Williams *v.* Beaumont and Duke has been stayed on appeal by Mr. Justice Wills and Mr. Justice Collins. Mr. Williams became a pauper inmate of the Lewisham Union Workhouse in 1893, being then in ill-health. He showed such evident marks of lunacy that Drs. Beaumont and Duke, the medical officer and assistant medical officer of the union, certified him as a lunatic, and he was removed to the Cane Hill Asylum, after having been three days detained under supervision in the workhouse. Williams was kept at Cane Hill for a month, and then discharged as not being insane, and not having shown any signs of insanity. Whereupon an action was commenced against the doctors for wrongful certification and detention, and want of due care in the exercise of their profession. The doctors appealed against the action, and it has now been stayed by order of the Court. The facts, however, are very simple, and, as the judges said, entirely exonerate the doctors from all blame. Mr. Williams was taken to the workhouse ill; but ill from what? From "alcoholism" the judges decided. His sister-in-law stated that he was given to drink. Moreover, he had threated to murder Mrs. Williams, and to commit suicide. If those were not proofs of insanity, what can be considered proofs? The real point of interest to doctors and lawyers is, whether or not a man suffering from alcoholism and delirium tremens is sane. Most certainly he is not sane, and most certainly any medical certificate affirming his insanity would be a mere scientific statement of the actual facts, just as a certificate affirming that a man was suffering from headache after a drinking bout would be. When a man is a lunatic he is a lunatic, whether his lunacy lasts three hours or three years; and a person suffering from delirium tremens is as certainly a lunatic whilst the delirium lasts as another man is a victim to headache whilst the headache lasts. If it be said that the victim of delirium tremens will, as a rule, recover in a few days if kept from drink, we admit it. But if he be a pauper, and have nobody to take care of him, who is to keep him from the drink? and who is to be responsible for his mad actions whilst he continues under the influence of drink and delirium? The speediest and much the best method of dealing with such a case is to hand the man over, during the stage of insanity, to those attendants who are accustomed to deal with insanity, exactly as we should hand over a case of small-pox to a small-pox nurse. When lunacy, acute or chronic, temporary or permanent, is recognised as a mere disease, all confusions of the kind here discussed will come to an end.

### Political Insanity.

Now that everybody is a politician the chances are increased a thousandfold that some politicians will become insane. It is well known that the mental balance is most easily upset by those subjects of thought which most deeply move the emotions. Love, religion, and politics are the three subjects which are recognised as the most exciting to the human mind. Religious mania is known to be one of the commonest of all forms of mania, so too is the mania of the sexual passion—erotomania. That political mania is not yet differentiated scientifically and legally is due to the fact that politics, until recently, have been largely the studies of a class. The case is different in our democratic times, when everybody is a politician, and the crudest minds take the keenest interest in the subject. Nothing better illustrates the truth of these propositions than the book just given to the world by Mr. J. P. Tynan on "The Irish Invincibles and their Times." Mr. Tynan is the notorious No. 1 of Parnellite days. He tells us to-day, in what he no doubt considers his sober moments, that when Lord Frederick Cavendish and Mr. Burke were murdered in Phœnix Park, "leading Irishmen over the world secretly rejoiced, applauding heartily in private circles, and speaking with joyful delight over what they called the glorious news from Ireland." We do not believe this, any more than we believe that hundreds of thousands of people in this country are ready to turn anarchists if opportunity should offer. What we do believe is, that men like Mr. Tynan and his class are genuinely insane, as we believe the *bonâ fide* anarchist to be insane. We would fain persuade politicians to accept this view. If they were convinced of it, the course of democratic politics in the near future would be greatly simplified. Common sense, common honesty, the uplifting of the many by the patient guidance of the few, would once more be the trusted and trustworthy guides of politicians of all ranks. The doctor has long played a conspicuous part in individual and family life. He and his science of mind and body must now be placed at the service of the public. When that is done, we may hope for sane politicians as well as sane private individuals, and that insane politicians will be kindly but firmly placed under such restraint as the public safety demands.

